# Dynamic transcriptome analysis identifies genes related to fatty acid biosynthesis in the seeds of *Prunus pedunculata* Pall

**DOI:** 10.1186/s12870-021-02921-x

**Published:** 2021-03-24

**Authors:** Wenquan Bao, Dun Ao, Lin Wang, Zhihao Ling, Maoshan Chen, Yue Bai, Ta-Na Wuyun, Junxing Chen, Shuning Zhang, Fengming Li

**Affiliations:** 1 Inner Mongolia Agricultural University, Hohhot, 010018 China; 2 State Key Laboratory of Tree Genetics and Breeding, Non-timber Forest Research and Development Center, Chinese Academy of Forestry, Zhengzhou, 450003 China; 3 Chengdu Jiyu Technology, Chengdu, 610213 Sichuan China; 4 Australian Center for Blood Diseases, Central Clinical School, Monash University, Melbourne, Victoria 3004 Australia

**Keywords:** Fatty acid, Oleic acid, Oil accumulation, Developing seeds, *Prunus pedunculata*, Transcriptome

## Abstract

**Background:**

*Prunus pedunculata* Pall, the deciduous shrub of *Amygdalus* subgenus in *Rosaceae*, is a new kind of desert oil-bearing tree. It has a long story of being planted in the West and North of China for sand fixation and desert control. In addition, the seeds of *P. pedunculata* are rich of oil, especially the monounsaturated fatty acid and polyunsaturated fatty acid. However, little is known about the molecular mechanisms of oil accumulation during the seed development of *P. pedunculata*.

**Results:**

The seeds of *P. pedunculata* from three independent plants at 10, 18, 24, 31, 39, 45, 59 and 73 days after flowering (DAF) were obtained and the oil compositions were evaluated. It showed that oleic acid was the dominant type of oil content in the mature seeds (from 32.724% at 10DAF to 72.06% at 73DAF). Next, transcriptome sequencing for the developing seeds produced 988.795 million high quality reads and TRINITY assembled 326,271 genes for the first transcriptome for *P. pedunculata*. After the *assembled* transcriptome was evaluated by BUSCO with 85.9% completeness, we identified 195,342, 109,850 and 121,897 *P. pedunculata* genes aligned to NR, GO and KEGG pathway databases, respectively. Then, we predicted 23,229 likely proteins from the assembled transcriptome and identified 1917 signal peptides and 5512 transmembrane related proteins. In the developing seeds we detected 91,362 genes (average FPKM > 5) and correlation analysis indicated three possible development stages – early (10 ~ 24DAF), middle (31 ~ 45DAF) and late (59 ~ 73DAF). We next analyzed the differentially expressed genes (DEGs) in the developing seeds. Interestingly, compared to 10DAF the number of DEGs was increased from 4406 in 18DAF to 27,623 in 73DAF. Based on the gene annotation, we identified 753, 33, 8 and 645 DEGs related to the fatty acid biosynthesis, lipid biosynthesis, oil body and transcription factors. Notably, *GPAT*, *DGD1*, *LACS2*, *UBC* and *RINO* were highly expressed at the early development stage, *ω6-FAD*, *SAD*, *ACP*, *ACCA* and *AHG1* were highly expressed at the middle development stage, and *LACS6*, *DGD1*, *ACAT1*, *AGPAT*, *WSD1*, *EGY2* and oleosin genes were highly expressed at the late development stage.

**Conclusions:**

This is the first time to study the developing seed transcriptome of *P. pedunculata* and our findings will provide a valuable resource for future studies. More importantly, it will improve our understanding of molecular mechanisms of oil accumulation in *P. pedunculata*.

**Supplementary Information:**

The online version contains supplementary material available at 10.1186/s12870-021-02921-x.

## Background

*Prunus pedunculata* Pall (*P. pedunculata*), belongs to the deciduous shrub of *Amygdalus* subgenus in *Rosaceae*, is a new kind of desert oil-bearing tree, which is also known as wild cherry, almond with stem and hairy cherry [1]. This rare and endangered wild tree species is naturally distributed in the mountainous and desert areas of arid and semi-arid areas in the Northwest China. Due to its strong adaptability, disease and insert resistance, cold and drought resistance, developed root system and long survival period, *P. pedunculata* has a long history of being planted in the West and North of China for sand fixation and desert control [2]. In addition, the *P. pedunculata* seeds are rich in protein and oil. The monounsaturated fatty acid and polyunsaturated fatty acid have been reported to be 69.11% and 28.77%, respectively, in the seeds of *P. pedunculata* [3]. However, much is unknown about *P. pedunculata* and their seeds.

The oil formation process in plants consists of four steps, including i) fatty acid de novo synthesis, ii) acyl elongation and editing, iii) triacylglycerol (TAG) assembly and iv) oil drop formation [4]. Several pathways, genes and proteins have been reported to be involved in the oil formation process. For example, the synthesis of fatty acid is localized to plastid while the assembly of TAG molecule occurs outside the plastid and is associated with the oil body [5]. The assembly of fatty acids occur on the ACP (acyl carrier protein) through a cycle of four reactions which elongate the acyl chain by 2 carbons and a total of 7 cycles are required to form the saturated 16 carbon acyl-ACP [6]. The content of fatty acid subtypes are decided by the activities of FATA (acyl-ACP thioesterase A), FATB (acyl-ACP thioesterase B), 18:0-ACP desaturase (SAD) and KASII (β-ketoacyl ACP synthase II) [6]. Although large is unknown about the transport of free fatty acid products from the plastid, it is implicated that LACS (long chain acyl-CoA synthetase) on the outer plastid envelope may function in the formation of acy-CoA, which is the substrate for glycerolipid assembly [4]. The esterification of newly synthesized fatty acid to phosphatidylcholine is reported to occur at the plastid envelop via LPCAT (acyl-CoA:lysophosphatidylcholine acyltransferase) [7]. The assembly of TAG from G-3-P (glycerol-3-phosphate) involves some key enzymes, including GPAT (glycerol-3-phosphate acyltransferase), LPAAT (lysophosphatidic acid acyltransferase), PAP (phosphatidic acid phosphatase) and DGAT (diacylglycerol acyltransferase) [8]. Transcription factors (TFs) WRI1 (ethylene-responsive transcription factor WRI1) and LEC1 (nuclear transcription factor Y subunit B-9) has been reported to control the expression of more than 15 enzymes (e.g., pyruvate dehydrogenase), which are required for the synthesis of fatty acid and the determination of the oil content in plant seeds [9, 10].

Transcriptome sequencing has enabled the identification of genes involved in the seed development and their association with the oil content in plant seeds. Fei utilized the transcriptome sequencing for the five seed development stages of *Zanthoxylum bungeanum* and identified 20 genes related to the fatty acid synthesis, such as *ENR*, *ECR* and *SAD1* [11]. Abdullah performed transcriptome sequencing for the developing seeds of *Camelina sativa* and identified 7932 genes involved in the triacylglycerol biosynthesis and accumulation [12]. Feng assembled the transcriptome for *Eucommia ulmoides* and reported 65 genes involved in fatty acid biosynthesis including *FABG* (3-oxoacyl-ACP reductase), *KASII* and *FABI* (enoyl-ACP reductase I) [13]. Yang reported 124 genes (e.g., *GmABI3b*, *GmNFYA*, *GmFAD2-1B*) potentially affecting the soybean oil content by analyzing the dynamic transcriptome of developing soybean seeds [14]. Li analyzed the de novo transcriptome of developing tree peony seeds and identified 388 genes (e.g., *SAD*, *FAD2*, *FAD8*) that might be involved in de novo fatty acid and TAG biosynthesis [15]. In addition, Wang identified 211 genes and 35 proteins associated with the fatty acid metabolism pathway, 63 genes and 11 proteins associated with the biosynthesis of unsaturated fatty acids, and 115 genes and 24 proteins associated with ALA (alpha-linolenic acid) metabolism in the tree peony seeds [16]. Kim identified 540 unique perilla genes involved in all known pathways of acyl-lipid metabolism by analyzing the transcriptome of seeds and leaves of *Perilla frutescens* [17]. However, little is known about the gene changes and their association with fatty acid synthesis during the seed development of *P. pedunculata* seeds.

In the present study, we examined the oil content of seven fatty acid subtypes in the *P. pedunculata* seeds at eight developing stages and performed transcriptome sequencing. We assembled the transcriptome for *P. pedunculata* seeds and annotated them. Then, differentially expressed genes (DEGs) were identified during the seed development and they might be related to the fatty acid synthesis. Weighted gene co-expression network analysis (WGCNA) revealed key genes for specific time points of the seed development and quantitative RT-PCR confirmed the expression changes of key genes involved in the seed development and oil accumulation in *P. pedunculata* seeds. This is the first time to study the transcriptome of *P. pedunculata* seeds and our study will provide a valuable resource for future studies related to *P. pedunculata*. The output of this study will improve our understanding towards the seed development and provides the molecular basis of oil accumulation in plants.

## Results

### Dynamic changes of oil content in the developing seeds of *Prunus pedunculata*

**Fig. 1:**
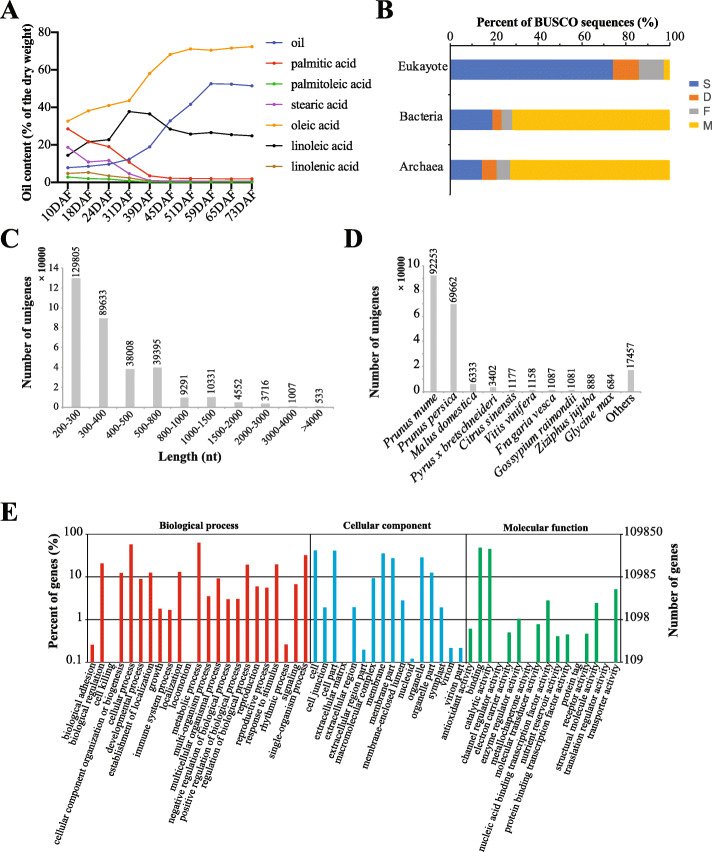
Oil content in the developing seeds of *P. pedunculata* and overview of the assembled transcriptome. **a** Proportions of different oil types in the developing seeds of *P. pedunculata* at 10, 18, 24, 31, 39, 45, 59 and 73 DAF. Error bars showing the standard variance are too small to be seen in the plot. **b** Evaluation of the assembled transcriptome using BUSCO. S: complete and single-copy BUSCOs; D: complete and duplicated BUSCOs; F: fragmented BUSCOs; M: missing BUSCOs. **c** Length distribution of the assembled genes for *P. pedunculata* seeds. **d** Number of genes aligned to different species in NR mapping results. **e** GO annotation for the assembled genes

The seeds of *P. pedunculata* were obtained from three plants at 10, 18 24, 31, 39, 45, 59 and 73 DAF. Then, using the Gas Chromatograph analysis we evaluated the oil content (Fig. 1a), including oil, palmitic acid, palmitoleic acid, stearic acid, oleic acid, linoleic acid and linolenic acid. It is notable that oleic acid was the major type of oil content and was gradually increased during the seed development from 32.724% (10DAF) to 72.060% (73DAF). The composition of linoleic acid and oil peaked at 31 (37.744%) and 59 (52.62%) DAF, respectively. Whereas the oil content remained ~ 50% after 59DAF. The other oil contents including palmitic acid, palmitoleic acid, stearic acid and linolenic acid were decreased during the seed development. This information provides a basis of understanding the oil content in the developing seeds of *P. pedunculata*.

### Transcriptome sequencing and de novo assembly

To study the gene changes and explore the molecular mechanisms in the developing seeds of *P. pedunculata*, we employed the transcriptome sequencing for all the samples mentioned above and three biological replicates were used. Initially, 988.795 million high quality reads were produced for the 24 samples (average = 41.20 million reads) by the transcriptome sequencing after data cleaning (Table 1). Then, we randomly selected one sample from the three replicates for de novo analysis. TRINITY assembled a total of 402,741 transcripts derived from 326,271 genes (Table 1). The GC content, N10, N20 and N50 of the assembled transcriptome were calculated as 40.55%, 2333, 1542, 552, respectively (Table 1). The total bases assembled for the developing seeds of *P. pedunculata* was 205.66 M and CD-HIT identified 198,113 clusters among the assembled transcriptome (Table 1) [18]. Next, we selected the longest transcripts as the unigene dataset and evaluated the completeness of the assembled unigenes using BUSCO [19]. Figure 1b showed that the assembled transcriptome had more similarity to eukaryote sequences, compared to archaea and bacteria sequences. We identified 219 (85.9%) complete BUSCOs out of the 255 total BUSCOs, including 189 (74.1%) complete and single-copy and 30 (11.8%) complete duplicated BUSCOs. Length distribution of the assembled transcriptome showed that 93.83% of the assembled unigenes were in the length 200 ~ 1000 nt and 533 (0.16%) unigenes were longer than 4000 nt (Fig. 1c).

**Table 1:** Overview of the assembled transcriptome of *P. pedunculata*

Category	Value
High quality reads	988,795,320
Average reads (high quality)	41,199,805
TRINITY transcripts	402,741
TRINITY genes	326,271
CD-HIT clusters	193,113
GC (%)	40.55
N10	2333
N20	1542
N50	552
Total assembled bases	205,656,558

### Transcriptome annotation

Next, we annotated the assembled seed transcriptome (326,271 unigenes) of *P. pedunculata* using multiple tools and databases. First, the transcriptome was aligned against the NCBI non-redundant (NR) and we found that 195,342, unigenes mapped. The NR mapping results showed that the top two hit species by the assembled unigenes were *Prunus mume* (92,253 unigenes) and *Prunus persica* (69,662 unigenes) (Fig. 1d). Second, we identified 109,850 unigenes aligned to the Gene Ontology (GO) database. GO annotation showed that 132,608, 118,239 and 80,695 unigenes were involved in the biological processes of “cellular process”, “metabolic process” and “single-organism process”, respectively (Fig. 1e). We also identified 65,847 and 64,407 unigenes related to “cell” and “cell part”, respectively (Fig. 1e), which might be related to the fatty acid synthesis. Third, we annotated the assembled transcriptome using the KEGG pathway database and found 121,897 unigenes aligned. It showed that 26,474, 14,804 and 2834 unigenes were involved in the pathways of “metabolic pathways” (ko01100), “biosynthesis of secondary metabolites” (ko01110) and “plant hormone signal transduction” (ko04075), respectively.

Then, Trinotate was used to further annotate the assembled developing seed transcriptome of *P. pedunculata*. Among the assembled transcriptome RNAMMER predicted 16 unigenes encoding ribosomal RNAs. Next, using TransDecoder we predicted 23,229 likely proteins produced by the assembled transcriptome. We found 18,002 (77.58%) and 16,120 (69.40%) of the likely proteins aligned to the UniProt/SwissProt and Pfam databases, respectively. From the likely proteins, SignalP and TmHMM identified 1917 signal peptides and 5512 transmembrane related proteins, respectively.

### Gene expression profiles and differential expression analysis

**Fig. 2:**
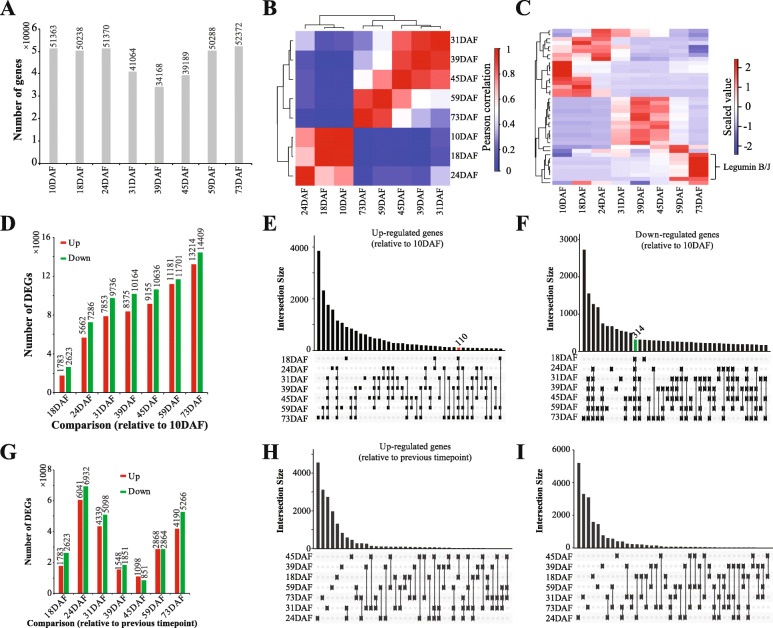
Gene expression profiles and differential expression analysis. **a** Number of genes identified in the *P. pedunculata* seeds at each developing stage. **b** Pearson correlation of the samples based on the gene expression profiles. **c** Hierarchical cluster analysis of samples based on the gene expression profiles. **d** Number of DEGs identified in the developing seeds of *P. pedunculata* compared to 10DAF. **e** Comparison of up-regulated genes in the developing seeds of *P. pedunculata* compared to 10DAF. (F) Comparison of down-regulated genes in the developing seeds of *P. pedunculata* compared to 10DAF. **g** Number of DEGs identified in the developing seeds of *P. pedunculata* compared to previous timepoint. **h** Comparison of up-regulated genes in the developing seeds of *P. pedunculata* compared to previous timepoint. **i** Comparison of down-regulated genes in the developing seeds of *P. pedunculata* compared to previous timepoint

We next aligned the clean reads to the assembled unigenes using Bowtie2 and profiled the gene expression for all samples using the RSEM method [20, 21]. We used FPKM (fragments per million reads per kilo base mapped) method to normalize the gene expression and filtered lowly expressed genes (average FPKM < 5) in each sample. As a result, for the developing seeds we identified a total of 91,362 genes, of which 51,363, 50,238, 51,370, 41,064, 34,168, 39,189, 50,288 and 52,372 distributed in 10DAF, 18DAF, 24DAF, 31DAF, 39DAF, 45DAF, 59DAF and 73DAF, respectively (Fig. 2a). We next analyzed the correlation between samples using the gene expression profiles. Figure 2b revealed that the seed development of *P. pedunculata* can be divided into three stages – early (10 ~ 24 DAF), middle (31 ~ 45 DAF) and late (59 ~ 73 DAF). Interestingly, highly expressed genes (top 10) of the *P. pedunculata* developing seeds confirmed these three development stages (Fig. 2c). Among the highly expressed genes, we found genes encoding proline-rich cell wall proteins specific to early developing seeds (10DAF and 18 DAF), genes encoding YSL9-like proteins specific to early developing seeds (10 ~ 24 DAF), and genes encoding legumin proteins specific to late developing seeds (31 ~ 73 DAF).

Next, we identified DEGs in the developing seeds of *P. pedunculata*. Using 10DAF as control, the numbers of DEGs identified in other samples (Additional file 1) can be seen in Fig. 2d. It is interesting that the numbers of DEGs were increased during the seed development, from 4406 DEGs in 18DAF to 27,623 in 73DAF. Notably, comparison of DEGs in the developing seeds of *P. pedunculata* revealed that 110 up-regulated (Fig. 2e) and 314 down-regulated (Fig. 2f) genes were shared by all the time points compared to 10DAF. Next, we analyzed the DEGs in the developing seeds compared to the previous timepoint. Figure 2g showed that the most DEGs were found in 24DAF vs 18DAF, followed by 73DAF vs 59DAF and 31DAF vs 24DAF. Comparison of DEGs identified in the developing seeds relative to the previous timepoint also confirmed that most DEGs identified in these time points and that no DEGs were identified to be up-regulated (Fig. 2h) or down-regulated (Fig. 2i) during the seed development.

### Fatty acid related genes

Based on the gene annotation for the developing seeds transcriptome of *P. pedunculata*, we identified 1246, 51, 9 and 2490 genes related to the fatty acid biosynthesis, lipid biosynthesis, oil body and TFs, respectively (Table 2). During the seed development, we identified 753, 33, 8 and 645 DEGs related to the fatty acid biosynthesis, lipid biosynthesis, oil body and TFs, respectively (Table 2).

**Table 2:** Number of genes related to the oil content in the seeds of *P. pedunculata*

Comparison	Fatty acid	Lipid	Oil body	TFs
Total gene	1246	51	9	2490
Total DEG	753	33	8	645
Compared to 10DAF:				
18DAF_vs_10DAF	15:51^a^	2:0	0:0	20:43
24DAF_vs_10DAF	85:132	1:2	1:0	107:86
31DAF_vs_10DAF	136:176	6:2	3:0	132:121
39DAF_vs_10DAF	168:190	5:7	3:0	140:124
45DAF_vs_10DAF	169:185	10:5	4:0	145:135
59DAF_vs_10DAF	163:205	14:8	4:0	183:92
73DAF_vs_10DAF	175:268	12:8	4:0	207:162
Compared to the previous timepoint:				
18DAF_vs_10DAF	15:51	2:0	0:0	20:43
24DAF_vs_18DAF	91:105	0:7	1:0	125:76
31DAF_vs_24DAF	79:93	6:3	3:0	76:72
39DAF_vs_31DAF	26:24	0:4	2:0	31:20
45DAF_vs_39DAF	17:16	7:0	2:0	20:18
59DAF_vs_45DAF	34:90	8:2	3:0	75:42
73DAF_vs_59DAF	52:105	2:6	0:5	54:103

^a^Numbers at the left and right sides of the colon represent the up-regulated and down-regulated genes

#### Fatty acid related pathways

**Fig. 3:**
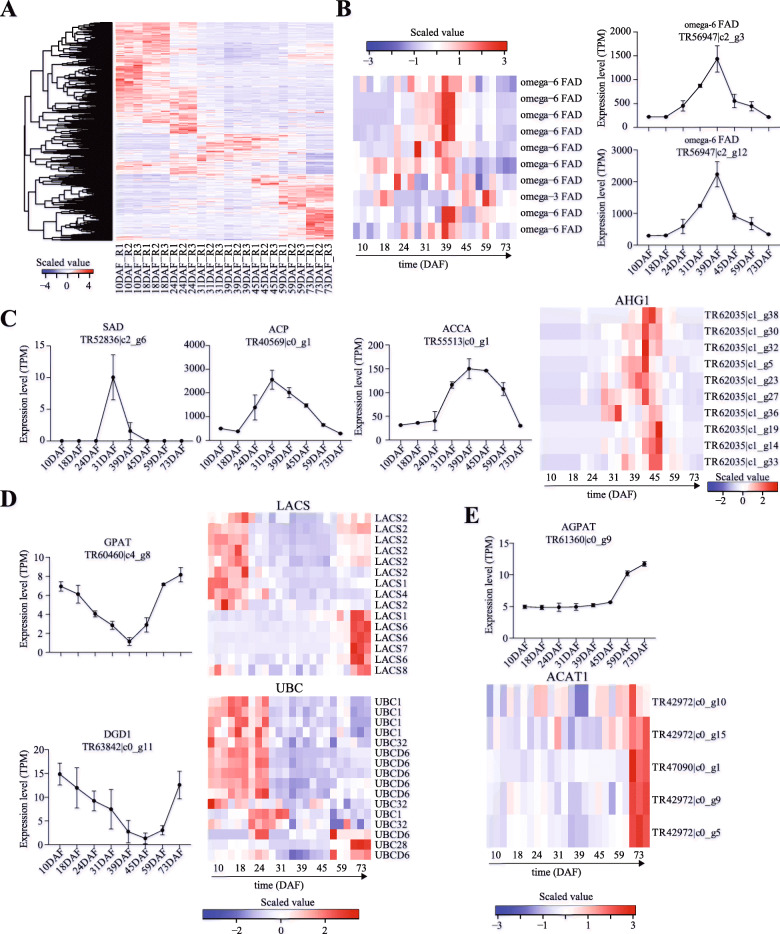
DEGs related to fatty acid pathways. **a** Hierarchical clustering of the fatty acid pathway associated DEGs identified in the developing seeds of *P. pedunculata*. **b** Heat map for differentially expressed FAD genes in the samples. **c** Expression levels of SAD, ACP and AHG1 genes in the developing seeds. **d** Expression of GPAT, ACCA, DGD1, LACSs and UBC genes in the developing seeds. **e** Expression patterns of AGPAT and ACAT1 genes in the developing seeds

In the seed development of *P. pedunculata* we identified 753 DEGs (Additional file 2) related to 24 fatty acid pathways/biological processes, including fatty acid metabolism (ko01212), biosynthesis of unsaturated fatty acids (ko01040), fatty acid biosynthesis (ko00061) and fatty acid transport (GO:0015908). The numbers of DEGs identified for each pathway/biological process can be found in Additional file 3 and we used a heat map (Fig. 3a) to show the expression levels of these 753 genes during the seed development. Interestingly, the highly expressed fatty acid related genes varied during the seed development. Thus, we examined the expression of DEGs encoding *FAD* (fatty acid desaturase), *SAD6* (stearoyl-[acyl-carrier-protein] 9-desaturase 6), *ACP* (acyl carrier protein 1), *ACCA* (acetyl-coenzyme A carboxylase carboxyl transferase subunit alpha), *LACS* (long chain acyl-CoA synthetase), *ACAT1* (acetyl-CoA acetyltransferase, cytosolic 1), *DGD1* (digalactosyldiacylglycerol synthase 1), *GPAT* (glycerol-3-phosphate acyltransferase), *AGPAT* (1-acyl-sn-glycerol-3-phosphate acyltransferase 1), *AHG1* (Probable protein phosphatase 2C 75) and *UBC* (ubiquitin-conjugating enzyme) during the seed development. According to their expression patterns during the seed development, these genes can be divided into three groups. The first group of DEGs consisted of *FAD*, *SAD6*, *ACCA* and *ACP*, which peaked at the middle stage of the seed development of *P. pedunculata* (Fig. 3b, c). Interestingly, we found that genes encoding omega-6 FAD peaked at 39DAF and that the omega-3 FAD gene peaked at 59DAF (left panel of Fig. 3b). The top two highly expressed genes encoding omega-6 FAD were TR56947|c2_g3 and TR56947|c2_g12 (right panel of Fig. 3b). The *SAD6* was shown to be detectable between 31DAF and 39DAF (Fig. 3c) and the *ACP* (TR40569|c0_g1) peaked at 31DAF (Fig. 3c). The second group included *GPAT*, *DGD1*, *LACS* and *UBC*, which were highly expressed at both early and late stages of the seed development (Fig. 3d). Interestingly, we found that *LACS2s* were highly expressed at early stage while other *LACS* (e.g., *LACS6*, *LACS7* and *LACS8*) were overexpressed at late stage of the seed development. Like *LACS* genes, *UBC1/UBC6* and *UBC28* were overexpressed at early and late stages, respectively (Fig. 3d). The third group include *AGPAT* and *ACAT1* genes, which were highly expressed at late stage of the seed development (Fig. 3e). The expression of *AGPAT* and *ACAT1* started to be increased at 45DAF and 73DAF, respectively.

#### Lipid biosynthesis

**Fig. 4:**
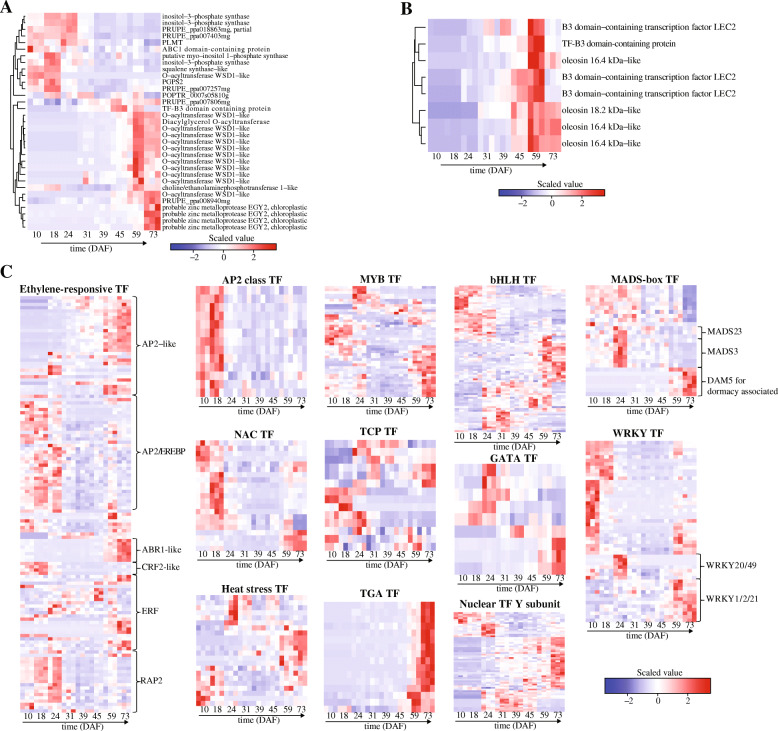
Heat maps of lipid biosynthesis, oil body and TF genes in the developing seeds of *P. pedunculata*. **a** Heat map of DEGs involved in the lipid biosynthesis. **b** Heat map of DEGs involved in oil body. **c** Heat maps of differentially expressed TF genes

Among the 55 genes involved in the lipid biosynthesis, 33 genes were differentially expressed during the seed development of *P. pedunculata* (Additional file 4). Interestingly, their expression patterns during the seed development were similar to *G3P*, *GPAT*, *LACS* and *UBC* (Fig. 4a). We found that *RINO*s (inositol-3-phosphate synthase) were highly expressed at the early stage and gene encoding TF-B3 domain-containing protein peaked at 45DAF (Fig. 4a). While another two groups of DEGs were highly expressed at the late stage of seed development, such as the O-acyltransferase WSD1-like genes, which peaked at 59DAF, and the *EGY2s*, which peaked at 73DAF.

#### Oil body biosynthesis

There were nine genes involved in the seed oil body biosynthesis process (GO:0010344) in the seeds of *P. pedunculata* (Table 2) and we identified 8 of them differentially expressed during the seed development (Additional file 5). Interestingly, their expression was found to be increased after 45DAF and peaked at 59DAF (Fig. 4b). Further, we found that the DEGs encoding *LEC2* TFs and TF-B3 domain containing protein were highly expressed at 59DAF and that DEGs encoding oleosins were highly expressed at 73DAF.

#### Transcription factors

Next, we would like to know the expression changes of TF genes and their relationship with the oil content during the seed development. As shown in Table 2, a total of 2490 TFs were annotated for *P. pedunculata* seeds and 645 were differentially expressed during the seed development (Additional file 1). Among them, 126 AP2-like/ER (ethylene-responsive), 12 *AP2*, 8 *GATA*, 26 *MADS-box*, 42 *MYB*, 20 *NAC*, 13 *TCP*, 15 *TGA*, 50 *WRKY*, 78 *bHLH*, 21 heat stress and 59 nuclear Y subunit TFs were identified (Additional file 1). Notably, most *AP2s* were highly expressed at the early stage while the TGA TFs were highly expressed at the late stages of the seed development (Fig. 4c). We also found that the ER TF subtypes may function at different stages of the seed development (Fig. 4c). For example, AP2/EREBP, CRF2-like, RAP2, ER may function in the initial stage; and AP2-like and ABR1-like, ER function at the late stage of the seed development. Like *ER*, *NAC*, *WRKY* were overexpressed at early and late stages (Fig. 4c). In addition, some TF subtypes were found to function during the whole process of the seed development, such as *MYB*, *TCP*, *GATA*, *bHLH*, heat stress, nuclear TF Y subunit and MADS-box (Fig. 4c). It is notable that *MADS-box* – *MADS23* and *MADS3* peaked at 24DAF while *DAM5* for dormancy associated *MADS-box* peaked at 59DAF and 73DAF (Fig. 4c). *WRKY20* and *WRKY49* were found to be overexpressed at 24DAF while genes encoding *WRKY1*, *WRKY2* and *WRKY21* were highly expressed at late stage of the seed development (Fig. 4c).

### Weighted gene co-expression network analysis

**Fig. 5:**
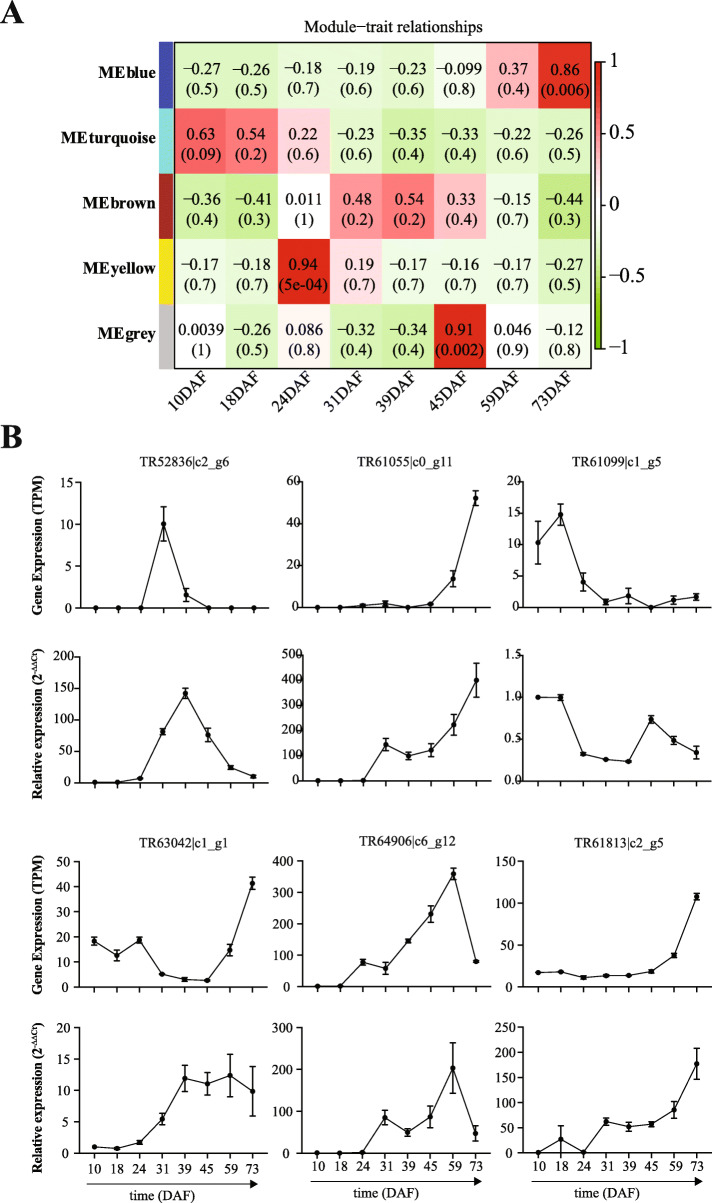
WGCNA and qRT-PCR. **a** WGCNA analysis identified co-expressed genes associated with the fatty acid biosynthesis. **b** qRT-PCR validation for six genes. For each gene the upper and lower panel of plots showed the expression levels (normalized to 10DAF) detected by transcriptome sequencing and qRT-PCR, respectively

To further investigate the association between DEGs and the oil content in the seeds of *P. pedunculata*, we performed the weighted gene co-expression network analysis (WGCNA). As a result, we identified 450, 133, 6, 743 and 103 genes from blue, brown, grey, turquoise and yellow modules, respectively (Fig. 5a, Additional file 6). It is notable that the blue module genes were associated with the late stage of seed development and the oil content in the seeds of *P. pedunculata*. Among the blue module genes, 420 were co-expressed with the oil, such as *ACOX1* (acyl-CoA oxidase 1), *ACOX4*, *LACS6*, oleosin, O-acyltransferase WSD1-like and various TFs (e.g., AP2-like ER, TGA). Interestingly, the other fatty acid types, including palmitic acid, palmitoleic acid, stearic acid, oleic acid, linoleic acid and linolenic acid, were found to be associated with the turquoise module genes (Additional file 6), which were significant at the early stage of the seed development. In addition, we found that the *omega-3 FAD* gene was co-expressed with the oil at 73DAF and that *SAD6* genes were significantly correlated with the *omega-6 FAD* genes during the middle seed development stage of *P. pedunculata*.

### Fatty acid biosynthesis pathway for *P. pedunculata* seed

**Fig. 6:**
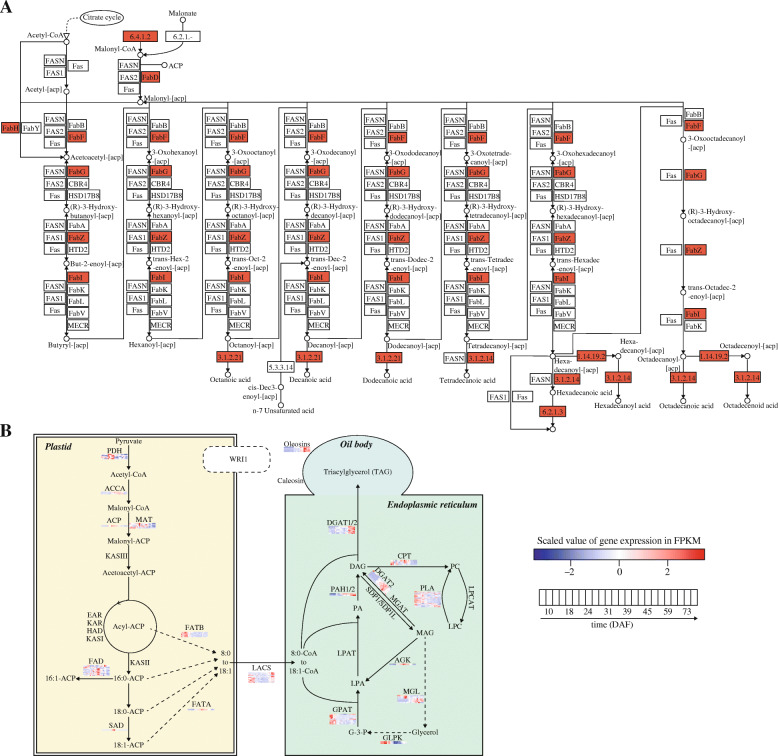
Proposed fatty acid biosynthesis pathway for *P. pedunculata* seeds. **a** General view of the fatty acid biosynthesis pathway based on the KEGG pathway annotation. The blocks in red represent the genes differentially expressed during the seed development of *P. pedunculata*. **b** Working model for the genes involved in the fatty acid and TAG biosynthesis in *P. pedunculata*. Heat maps represent the expression levels (scaled values) of genes in the developing seeds of *P. pedunculata*. DAG: 1,2-Diacylglycerol; PA: phosphatidic acid; LPA: lysophosphatidic acid; G-3-P: glycerol-3-phosphate; PC: phosphatidylcholine; LPC: lysophosphatidylcholine, and glycerol; MAG: monoacylglycerol; PDH: pyruvate dehydrogenase complex; ACCA: acetyl-coenzyme A carboxylase carboxyl transferase subunit alpha; ACP: acyl carrier protein; MAT: malonyl-CoA-acyl carrier protein transacylase; KAS: ketoacyl-ACP synthase; EAR: enoyl-ACP reductase; KAR: ketoacyl-ACP reductase; HAD: hydroxyacyl-ACP dehydrase; FAD: fatty acid desaturase; SAD: 18:0-ACP desaturase; FATB: acyl-ACP thioesterase B; FATA: acyl-ACP thioesterase A; DGAT: diacylglycerol acyltransferase; PAH: phosphatidic acid phosphohydrolase; LPAT: acyl-CoA:acylglycerol-3-phosphate acyltransferase; GPAT: glycerol-3-phosphate acyltransferase; CPT: diacylglycerol cholinephosphotransferase; MGAT: monoacylglycerol acyltransferase; SDP: suger-dependent protein; AGK: acylglycerol kinase; MGL: acylglycerol lipase; GLPK: glycerol kinase; PLA: phospholipase; LPCAT: lysophosphatidylcholine acyltransferase

To propose a proper pathway involved in the fatty acid biosynthesis in the developing seeds of *P. pedunculata*, we first highlighted the DEGs in the general fatty acid biosynthesis pathway based on the KEGG pathway annotation (Fig. 6a). It showed that some key genes of the fatty acid biosynthesis were identified in this study, including 6.4.1.2 (acetyl-CoA carboxylase, *ACACA*), 3.1.2.14 (fatty acyl-ACP thioesterase B, *FATB*), 3.1.2.21 (medium-chain acyl-[acyl-carrier-protein] hydrolase, *MCH*), 1.14.19.2 (acyl-[acyl-carrier-protein] desaturase, *FAB2*), 6.2.1.3 (long-chain acyl-CoA synthetase, *ACSL*), *FabD*, *FabF*, *FabH*, *FabI*, *FabG* and *FabZ*. Their products are either substrates, intermediates or important enzymes. Next, we proposed the working model of fatty acid synthesis in plastid and endoplasmic reticulum (Fig. 6b). In addition to the DEGs described above, we found extra important genes related to the fatty acid biosynthesis. For example, *PDH* (pyruvate dehydrogenase complex), involved in the overall conversion of pyruvate to acetyl-CoA, was found to be highly expressed in the seeds of 24DAF and 31DAF (Additional file 1). Some other genes from this model were also found to be differentially expressed in the developing seeds of *P. pedunculata*, such as *MAT* (malonyl-CoA-acyl carrier protein transacylase), *FATB*, *DGAT*, *PAH* (phosphatidic acid phosphohydrolase), *CPT* (diacylglycerol cholinephosphotransferase), *PLA* (phospholipase), *AGK* (acylglycerol kinase) and *GLPK* (glycerol kinase). However, some genes like *KASI* (ketoacyl-ACP synthase I), *KASII* (ketoacyl-ACP synthase II), *KASIII* (ketoacyl-ACP synthase III), *WRI*, *LPCAT* (lysophosphatidylcholine acyltransferase), *SDP* (sugar-dependent protein) and *LPAT* (acyl-CoA:acylglycerol-3-phosphate acyltransferase) were not detected in the developing seeds of *P. pedunculata*. The reason for their absence requires further experiments to be explored.

### qRT-PCR

Then, we performed quantitative real-time PCR to validate the gene expression changes during the seed development of *P. pedunculata*. A total of six genes (TR52836|c2_g6, TR61055|c0_g11, TR61099|c1_g5, TR61813|c2_g5, TR63042|c1_g1, TR64906|c6_g12) were randomly selected and the 18S rRNA was used as the internal control. The primers can be accessed in the Additional file 7 and nine reactions were performed for every gene at each time point of the seed development. Relative normalized expression (RNE) was used to show the expression of all genes in the samples (relative to 10DAF). As a result, we found that the expression patterns of all six genes were agreed by both RNA-Seq and qRT-PCR (Fig. 5b). It is notable that the high expression of TR61055|c0_g11 (transcription factor TGA2), TR63042|c1_g1 (MYB transcription factor 3) and TR61813|c2_g5 (long chain acyl-CoA synthetase 6, *LACS6*) were confirmed by qRT-PCR at the late seed development stage and the high expression of TR61099|c1_g5 (fatty acyl-ACP thioesterase B, *FATB*) was validated in the early seed development stage (Fig. 5b). From the sequencing and qRT-PCR results we found that TR64906|c6_g12 (B3 domain-containing transcription factor LEC2) peaked at 59DAF. TR52836|c2_g6 (stearoyl-[acyl-carrier-protein] 9-desaturase 6, *SAD6*) was found to be highly expressed at 31DAF by the sequencing and at 39DAF by qRT-PCR (Fig. 5b) and both time points are defined as in the middle seed development stage. The high agreement of gene expression patterns by transcriptome sequencing and qRT-PCR revealed that the genes identified in this study might be functional in the development and the oil content of the *P. pedunculata* seeds.

## Discussions

Seed oil content is a new important trait of *P. pedunculata*, however, little is known about the molecular basis of oil accumulation and genes involved in the oil synthesis in *P. pedunculata* seeds, probably due to the missing of its genome sequence. Here, we assembled the transcriptome for *P. pedunculata* seeds for the first time and studied the dynamic gene profiles of the seeds at eight developing points. We examined the content of different fatty acid types, including oil, palmitic acid, palmitoleic acid, stearic acid, oleic acid, linoleic acid and linolenic acid, in the developing seeds of *P. pedunculata* (Fig. 1a). The three major fatty acid types were oil, oleic acid and linoleic acid in the mature seeds of *P. pedunculata*. Interestingly, oleic acid was shown to be the highest type in the *P. pedunculata* seeds, and the proportion is similar to camellia and olive oil [22, 23]. The decrease of palmitic acid, palmitoleic acid, stearic acid and linolenic acid during the seed development might link with the high expression of *LACS2* at the early developing stage of *P. pedunculata* seeds (Fig. 1a, Fig. 3d). In *Arabidopsis* LACS2 enzyme preferers using palmitic acid and palmitoleic acid as substrates to form the cutin or cuticular waxes [24].

According to the gene expression profiles, the seed development of *P. pedunculata* can be divided into three stages (Fig. 2b, c). We observed that genes encoding *GPAT*, *DGD1*, *LACS2*, *UBC*, *RINO* and multiple TFs (e.g., *AP2/EREBP ER*, *CRF2-like*, *RAP2*, *AP2*, *NAC*, *MYB*, *MADS-box*, *WRKY*) were highly expressed at the early stage (10 ~ 24DAF) of the seed development (Fig. 3d, Fig. 4a, c). GPAT exhibits sn-1 acyltransferase activity with high specificity of acyl-CoAs, has the potential of triggering the biosynthesis of storage lipid and plays a pivotal role in the Kennedy pathway, the de novo assembly of TAG from G-3-P and acyl-CoAs and the glycerolipid biosynthesis [25]. The G-3-P is acylated by GPAT and LPAT to yield the phosphatidic acid, which is then hydrolyzed to form diacylglycerol, the final substrate for TAG via DGAT (diacylglycerol acyltranstransferase) [17]. The UBC enzymes are essential for the fatty acid β-oxidation during the early seedling development of oilseed plants [26]. The high expression of these genes at the early development stage might contribute to the accumulation for the fatty acid biosynthesis in subsequent processes.

At the middle stage of seed development (31 ~ 45DAF), we identified *ω6-FAD*, *SAD*, *ACP*, *ACCA*, *AHG1* and some TF (e.g., TCP, heat stress, bHLH, nuclear TF Y subunit) genes highly expressed (Fig. 3, Fig. 4). ACCA is the first enzyme involved in the pathway of fatty acid synthesis in plastid and regulates the flux of carbon into fatty acids [4]. Whereas the assembly of fatty acids usually happens on the ACP through a cycle of 4 reactions of elongating the acyl chain by 2 carbons each time [6]. The *FAD* genes have been described to correlate the seed development and the fatty acid biosynthesis in some oil plants, including *Zanthoxylum bungeanum* [11], *Camelina sativa* [12], *Linum usitatissimum* [27], *Eucommia ulmoides* [13], *Glycine max* [14] and *Paeonia section* [16]. *FAD2* and *FAD3* in the ER (endoplasmic reticulum) catalyze the desaturation of fatty acids attached to phospatidylcholine (PC) from PC-18:1 to PC-18:2 (*FAD2*) and from PC-18:2 to PC-18:3 (*FAD3*) [28–30]. *FAD6* has the potential of introducing the second double bond in the biosynthesis of 16:3 and 18:3 fatty acids, which are important constituents of plant membrane, and functions on fatty acids esterified to galactolipids, sulfolipids and phosphatidylglycerol through ferredoxin [31]. It is reported that stearic acid is converted into oleic acid (highest content in the *P. pedunculata* seeds) by *SAD*, which is one of the regulators for the proportion of fatty acids in seeds [4]. While AHG is a negative regulator of abscisic acid responses during the seed germination [32]. The high expression of key regulatory genes during the middle stage of seed development may indicate the oil storage and the determination of the contents of fatty acids.

Next, we would like to discuss highly expressed genes identified in the late stage (59 ~ 73DAF) of the seed development, such as *LACS6*, *DGD1*, *ACAT1*, *AGPAT*, *WSD1*, *EGY2* and oleosin (Fig. 3, Fig. 4). Using palitate, palmitoleate, oleate and linoleate as substrates, LACS6 has the potential of activating the long chain fatty acids for both synthesis of cellular lipid and degradation through beta-oxidation [33, 34]. DGD1 is specific for α-glycosidic linkages and involved in the synthesis of diacylglycerol galactolipids which are specifically localized in the thylakoid membranes [35, 36]. The overexpression of *ACAT1*, a CoA biosynthetic enzyme, has been shown to lead the increase of oil content in Arabidopsis [37]. AGPAT is a plastidial enzyme of the prokaryotic glycerol-3-phosphate pathway, which converts the lysophosphatidic acid into phosphatidic acid by incorporating an acyl moiety at position sn-2 [38]. The o-acyltransferase WSD1, the major wax ester synthase, has been reported to be involved in the drought tolerance in plants [39, 40]. In Camelina seeds the expression of *WSD1* was up-regulated during the early seed development and correlated with the oil content of the seeds [12]. Oleosin are the major proteins involved in the oil body biogenesis and the stabilization of TAG/cytosol oil body interface [41]. The expression of oleosin genes has been shown to be up-regulated during the seed development in plants, such as *Camelina sativa* [12] soybean [14], *Paeonia section* [15] and *Perilla frutescens* [17]. Overall, these genes have been shown to participate the fatty acid biosynthesis, however, their functions in the oil accumulation of *P. pedunculata* seeds are still not clear. Together with these studies, the high expression of these genes supports them to play a key role during the seed development and fatty acid biosynthesis in *P. pedunculata*i.

The fatty acid biosynthesis is a complicated process that involves many enzymes and molecules. It is the fundamental to the production of membranes and lipids in the plastids of plants [13]. We proposed a proper working model for the genes involved in the fatty acid biosynthesis for *P. pedunculata* (Fig. 6b). Some key genes from the process were identified with differential expression in the developing seeds of *P. pedunculata*. However, we missed some known important regulators that have been reported to participate in the fatty acid biosynthesis, such as *KASI* ~ *III* [13], *WRI1* [9] and *LPAT* [17]. There might be some explanations for their absence, including 1) the de novo assembly method or the annotation method missed these genes; 2) they were not detected by the transcriptome sequencing; and 3) they were not expressed in the seeds of *P. pedunculata*. Future studies need to be performed to complete the working model of fatty acid biosynthesis in *P. pedunculata*. In addition, we identified some TFs that might be involved in the fatty acid biosynthesis of *P. pedunculata*, however, the regulation mechanism during the oil accumulation is still not clear. Future experiments are required to investigate their functions in the fatty acid biosynthesis for *P. pedunculata*.

## Conclusions

In conclusion, we assembled the first transcriptome for the developing seeds (eight time points) of *P. pedunculata*. In total, 402,741 transcripts of 326,271 unigenes were reported with the GC ratio of 40.55%. Annotation of the transcriptome showed 109,850 and 121,897 unigenes aligned to the GO and KEGG pathway databases, respectively. We also predicted 23,229 likely proteins produced by the *P. pedunculata* seed transcriptome, of which 1246, 51, 9 and 2.490 related to fatty acid biosynthesis, lipid biosynthesis, oil body and TFs, respectively. We profiled 91,362 genes expressed more than 5 FPKM in the developing seeds of *P. pedunculata* and identified 48,788 DEGs. Compared to 10DAF, the numbers of DEGs were shown to be increased during the seed development, from 4406 in 18DAF to 27,623 in 73DAF. We found that 753, 33, 8 and 645 DEGs related to the fatty acid biosynthesis, lipid biosynthesis, oil body and TFs, respectively. *GPAT*, *DGD1*, *LACS2*, *UBC* and *RINO* were highly expressed at the early seed development (10 ~ 24DAF), *ω6-FAD*, *SAD*, *ACP*, *ACCA* and *AHG1* were highly expressed at the middle seed development (31 ~ 45DAF) while *LACS6*, *DGD1*, *ACAT1*, *AGPAT*, *WSD1*, *EGY2* and oleosin genes were abundant at the late seed development. The genes expression changes were confirmed by qRT-PCR. This is the first time to study the transcriptome of *P. pedunculata* developing seeds and our findings will provide a valuable resource for future studies. More importantly, it will improve our understanding of the oil accumulation for *P. pedunculata*.

## Methods

### Plant material

We selected three six-year-old plants of *Prunus pedunculata* Pall which were planted in the experimental fields of Inner Mongolia Breeding Center, China. The original seeds were obtained from the wild place of Baotou, China (110.066562,41.039184) and maintained in the Inner Mongolia Seed Museum as Mengbian-1. The days after flowering (DAF) of fruits were marked with tags to track the seed development. From 10th April of 2019, we collected the fruits of *Prunus pedunculata* at 10, 18, 24, 31, 39, 45, 59 and 73 DAF with three biological replicates (*n* = 3). Every development stage we collected a total of ~ 100 seeds at each timepoint and peeled off the peel and stone. Then, the nucleolus was flash frozen in liquid nitrogen and stored at − 80 °C until further use. As the ovules at 0 DAF were hard to collect, we used seeds at 10 DAF as the control.

### Quantitation of oil content

The oil content of *P. pedunculata* seeds was determined using the Gas Chromatograph (GC, Agilent 6890 N) according to the manufacturer’s protocol, as described [14]. The CP-Sil 88 (Agilent Technology) and nitrogen gas were used as the GC column and carrier gas, respectively. The initial temperature was set to 180 °C and raised to 200 °C at the rate 6 °C/min. After 3 min, the temperature was then raised to 240 at the rate 10 °C/min. The samples were ground with grinder at low temperature and then dried in the vacuum freezing dryer. Then, powder samples (100 mg) were diluted in 1% heptadecanoic acid (with internal standard) in 2 mL centrifuge tubes. After 1 mL methylation agent (2.5%, v/v, H2SO4 in CH3OH) was added to each tube, the samples were water bathed for the methyl esterification at 85 °C for 1 h. We collected the extractions, centrifuged them and retained the supernatants, followed by adding 600 μL NaCl (0.9%, w/v) and 350 μL n-hexane later. Then, the mixture was centrifuged at 4000 RPM for 10 min and the organic phase was air-dried. After 500 μL ethyl acetate was added to the dried methyl esterification samples, they were subjected to the GC for oil content analysis. Compared to the standard, the fatty acids were examined qualitatively and quantitatively by the peak area method. The absolute content of fatty acids was determined by the methyl ester standard. This experiment was replicated three times and average values were calculated for each oil type.

### Total RNA extraction, library construction and sequencing

Total RNA was extracted from the nucleolus using the TRIzol reagent (Invitrogen), as previously described [42]. After the quality and quantity of the total RNA were examined using the Agilent 2100 Bioanalyzer, equal amount of total RNA (1 μg) was used for the library construction at BGI-Shenzhen. In brief, poly(A) mRNAs were enriched and pulled down by the magnetic oligo (dT) beads and fragmented into small pieces (~ 200 bp). Then, random hexamer (N6) primers were used to construct the double strand cDNA libraries. After the end repair by phosphate at 5′-end and sticky “A” at 3′-end, the cDNA libraries were ligated with sequencing primers to build the final RNA-Seq libraries. Next, the final libraries were sequenced on the BGISEQ-500 RS platform with paired-end 150 strategy.

### De novo assembly

We used Trim_galore to remove low quality reads, reads with adapters and contamination reads from the raw reads. FASTQC (http://www.bioinformatics.babraham.ac.uk/projects/fastqc/) was used to evaluate the clean reads for quality control. Then, clean reads of each time point were subjected to the TRINITY software for de novo assembly, as described previously [43]. CD-HIT was used to evaluate the clusters of the transcripts [18]. Then, we selected the longest transcripts (used as unigene) using the program provided by Trinity and extracted the likely proteins using TransDecoder. To evaluate the completeness of the assembled unigenes, we employed BUSCO to map the unigenes to three datasets, including archaea, bacteria and eukaryota [19].

### Transcription annotation

We first annotated the assembled transcriptome using Trinotate, according to the protocol [44]. In brief, the unigenes and likely protiens were aligned against the UniProt/Sprot using BLASTX and BLASTP, respectively, with the e-value <1e-3. RNAmmer (v1.2) [45], HMMER [46], SignalP (v4.1) [47] and TMHMM Server (v.2.0) [48] were used to predict the rRNA transcripts, protein domains, signal peptides and transmembrane regions in the assembled unigenes and likely proteins. Then, all the annotations were subjected to Trinotate to generate a report.

Next, we aligned the assembled unigenes to NCBI non-redundant (NR), UniProt and Kyoto Encyclopedia of Genes and Genomes (KEGG) databases to obtain the Gene Ontology (GO) and pathway annotation, as previously described [43]. After the matched unigenes were selected (e-value <1e-5), BLAST2GO was used to retrieve associated GO items describing biological process (BP), cellular component (CC) and molecular function (MF). Unigenes with enzyme commission number (EC) were obtained and searched in the KEGG metabolic pathway database. The KEGG pathway annotation (release 90.0) for the assembled transcriptome was performed at BGI-Shenzhen [49].

### Gene expression profiles and differential expression analysis

We used Bowtie2 [20, 50] and RSEM (RNA-Seq by Expectation-Maximization) [21] to align the clean reads to the assembled unigenes and profile gene expression, respectively, for each sample. FPKM (fragments per million reads per kilo base mapped) method was used for normalization and average FPKM of each gene were calculated for each time point. After lowly expressed genes (average FPKM < 5) were filtered, we performed differential expression analysis using edgeR with following cut-offs: log2 fold change (log2FC) > 1 or < − 1, coefficient of variation (CV) < 0.7, *p-value* < 0.5 and false discovery rate (FDR) < 0.1 [50].

### Functional analysis

We performed functional analysis for the differentially expressed genes using the GO and KEGG pathway annotation results. To identify significantly enriched GO terms and pathways, we first calculated the *p-value* using the Fisher’s Exact Test to present the enrichment significance and calculated the corrected p-value (shown as q-value) using the qvalue package in R platform. Significantly enriched GO terms and KEGG pathways were selected as follows: *q-value* < 0.05 and non-plant associated terms/pathways were filtered.

### qRT-PCR

We performed quantitative real-time PCR (qRT-PCR) to validate the gene expression changes during the seed development of *Prunus pedunculata*. We randomly selected six genes (TR52836|c2_g6, TR61055|c0_g11, TR61099|c1_g5, TR61813|c2_g5, TR63042|c1_g1, TR64906|c6_g12) and used the 18S rRNA as the internal control. Forward and reverse primers were predicted for the candidate genes and control using the Beacon Designer 7.9. After the total RNA was extracted from the seeds at all eight time points (as described above), an aliquot (2 mg) of the total RNA was used for the first-strand cDNA synthesis using the TRUEScript First-Strand cDNA Synthesis kit (Aidlab Biotech, Beijing, China). Then, the cDNA (1 μL) was used to build the qRT-PCR reaction mixture (10 μL) together with 2 × SYBR Green Supermix (5 μL, DBI, China), forward primer (0.5 μL), reverse primer (0.5 μL) and ddH_2_O (3 μL). The qPCR mixture was run on the qTOWER 2.2 qRT-PCR Thermal Cyclers (Analytik Jena, Germany) following the protocols. Then, Ct value of each gene in each sample was obtained and ΔCt was calculated. The 10DAF samples were used as control and the expression levels of all genes in the other samples were calculated present using the relative normalized expression: *RNE* = 2^−ΔΔCt^. Three reactions were performed for each gene in every single replicate, thus we obtained nine replicates (*n* = 9) for each gene at each timepoint.

## Supplementary Information

**Additional file 1. **Differentially expressed genes in the developing seeds of *P. pedunculata*.

**Additional file 2. **Differentially expressed genes related to fatty acid in the developing seeds of *P. pedunculata*.

**Additional file 3.** Numbers of differentially expressed genes of fatty acid related pathway/biological process.

**Additional file 4. **Differentially expressed genes related to lipid biosynthesis in the developing seeds of *P. pedunculata*.

**Additional file 5. **Differentially expressed genes related to oilbody in the developing seeds of *P. pedunculata*.

**Additional file 6.** WGCNA identifies co-expressed genes and their relationship with the oil contents.

**Additional file 7.** Forward and reverse primers for qRT-PCR.

## Data Availability

The raw sequencing data can be accessed from the NCBI Sequence Read Archive (SRA) platform (http://trace.ncbi.nlm.nih.gov/Traces/sra/) under the accession number PRJNA684995.

## Abbreviations

DAF: Days after flowering
GO: Gene Ontology
TF: Transcription factor
FPKM: Fragments per million reads per kilo base mapped
TAG: Triacylglycerol
NR: NCBI non-redundant database
DEG: Differentially expressed gene

## References

[1] Li C, Yang J, Yao L, Qin F, Hou G, Chen B, Jin L, Deng J, Shen Y (2020). Characterisation, physicochemical and functional properties of protein isolates from Amygdalus pedunculata pall seeds. Food Chem.

[2] Chu J, Xu X, Zhang Y (2013). Production and properties of biodiesel produced from Amygdalus pedunculata pall. Bioresour Technol.

[3] Gao Y, Li C, Chen B, Shen YH, Han J, Zhao MG (2016). Anti-hyperlipidemia and antioxidant activities of Amygdalus pedunculata seed oil. Food Funct.

[4] Bates PD, Stymne S, Ohlrogge J (2013). Biochemical pathways in seed oil synthesis. Curr Opin Plant Biol.

[5] Chapman KD, Ohlrogge JB (2012). Compartmentation of triacylglycerol accumulation in plants. J Biol Chem.

[6] Li-Beisson Y, Shorrosh B, Beisson F, Andersson MX, Arondel V, Bates PD, Baud S, Bird D, Debono A, Durrett TP (2013). Acyl-lipid metabolism. Arabidopsis Book.

[7] Tjellstrom H, Yang Z, Allen DK, Ohlrogge JB (2012). Rapid kinetic labeling of Arabidopsis cell suspension cultures: implications for models of lipid export from plastids. Plant Physiol.

[8] Barron EJ, Stumpf PK (1962). Fat metabolism in higher plants. XIX. The biosynthesis of triglycerides by avocado-mesocarp enzymes. Biochim Biophys Acta.

[9] Cernac A, Benning C (2004). WRINKLED1 encodes an AP2/EREB domain protein involved in the control of storage compound biosynthesis in Arabidopsis. Plant J.

[10] Shen B, Allen WB, Zheng P, Li C, Glassman K, Ranch J, Nubel D, Tarczynski MC (2010). Expression of ZmLEC1 and ZmWRI1 increases seed oil production in maize. Plant Physiol.

[11] Fei X, Ma Y, Hu H, Wei A (2020). Transcriptome analysis and GC-MS profiling of key genes in fatty acid synthesis of Zanthoxylum bungeanum seeds. Ind Crop Prod.

[12] Abdullah HM, Akbari P, Paulose B, Schnell D, Qi W, Park Y, Pareek A, Dhankher OP (2016). Transcriptome profiling of Camelina sativa to identify genes involved in triacylglycerol biosynthesis and accumulation in the developing seeds. Biotechnol Biofuels.

[13] Feng Y, Wang L, Fu J, Wuyun T, Du H, Tan X, Zou F, Li F. Transcriptome sequencing discovers genes related to fatty acid biosynthesis in the seeds of Eucommia ulmoides. Genes & Genomics 2016;38(3):275-83.

[14] Yang S, Miao L, He J, Zhang K, Li Y, Gai J: Dynamic Transcriptome Changes Related to Oil Accumulation in Developing Soybean Seeds. Int J Mol Sci 2019;20(9):2202. 10.3390/ijms20092202 PMC6539092 31060266

[15] Li SS, Wang LS, Shu QY, Wu J, Chen LG, Shao S, Yin DD (2015). Fatty acid composition of developing tree peony (Paeonia section Moutan DC.) seeds and transcriptome analysis during seed development. BMC Genomics.

[16] Wang X, Liang H, Guo D, Guo L, Duan X, Jia Q, Hou X (2019). Integrated analysis of transcriptomic and proteomic data from tree peony (P. ostii) seeds reveals key developmental stages and candidate genes related to oil biosynthesis and fatty acid metabolism. Hortic Res.

[17] Kim HU, Lee KR, Shim D, Lee JH, Chen GQ, Hwang S (2016). Transcriptome analysis and identification of genes associated with omega-3 fatty acid biosynthesis in *Perilla frutescens* (L.) var. frutescens. BMC Genomics.

[18] Li W, Godzik A (2006). Cd-hit: a fast program for clustering and comparing large sets of protein or nucleotide sequences. Bioinformatics.

[19] Seppey M, Manni M, Zdobnov EM (1962). BUSCO: assessing genome assembly and annotation completeness. Methods Mol Biol.

[20] Langmead B, Salzberg SL (2012). Fast gapped-read alignment with bowtie 2. Nat Methods.

[21] Li B, Dewey CN (2011). RSEM: accurate transcript quantification from RNA-Seq data with or without a reference genome. BMC Bioinformatics.

[22] Wu B, Ruan C, Han P, Ruan D, Xiong C, Ding J, Liu S (2019). Comparative transcriptomic analysis of high- and low-oil Camellia oleifera reveals a coordinated mechanism for the regulation of upstream and downstream multigenes for high oleic acid accumulation. 3 Biotech.

[23] D'Angeli S, Altamura MM: Unsaturated Lipids Change in Olive Tree Drupe and Seed during Fruit Development and in Response to Cold-Stress and Acclimation. Int J Mol Sci 2016;17(11):1889. 10.3390/ijms17111889 PMC5133888 27845749

[24] Schnurr J, Shockey J, Browse J (2004). The acyl-CoA synthetase encoded by LACS2 is essential for normal cuticle development in Arabidopsis. Plant Cell.

[25] Singer SD, Chen G, Mietkiewska E, Tomasi P, Jayawardhane K, Dyer JM, Weselake RJ (2016). Arabidopsis GPAT9 contributes to synthesis of intracellular glycerolipids but not surface lipids. J Exp Bot.

[26] Theodoulou FL, Eastmond PJ (2012). Seed storage oil catabolism: a story of give and take. Curr Opin Plant Biol.

[27] Xie D, Dai Z, Yang Z, Tang Q, Deng C, Xu Y, Wang J, Chen J, Zhao D, Zhang S, Zhang S, Su J (2019). Combined genome-wide association analysis and transcriptome sequencing to identify candidate genes for flax seed fatty acid metabolism. Plant Sci.

[28] Sperling P, Heinz E (1993). Isomeric sn-1-octadecenyl and sn-2-octadecenyl analogues of lysophosphatidylcholine as substrates for acylation and desaturation by plant microsomal membranes. Eur J Biochem.

[29] Okuley J, Lightner J, Feldmann K, Yadav N, Lark E, Browse J (1994). Arabidopsis FAD2 gene encodes the enzyme that is essential for polyunsaturated lipid synthesis. Plant Cell.

[30] Browse J, McConn M, James D, Miquel M (1993). Mutants of Arabidopsis deficient in the synthesis of alpha-linolenate. Biochemical and genetic characterization of the endoplasmic reticulum linoleoyl desaturase. J Biol Chem.

[31] Falcone DL, Gibson S, Lemieux B, Somerville C (1994). Identification of a gene that complements an Arabidopsis mutant deficient in chloroplast omega 6 desaturase activity. Plant Physiol.

[32] Nishimura N, Yoshida T, Kitahata N, Asami T, Shinozaki K, Hirayama T (2007). ABA-hypersensitive Germination1 encodes a protein phosphatase 2C, an essential component of abscisic acid signaling in Arabidopsis seed. Plant J.

[33] Shockey JM, Fulda MS, Browse JA (2002). Arabidopsis contains nine long-chain acyl-coenzyme a synthetase genes that participate in fatty acid and glycerolipid metabolism. Plant Physiol.

[34] Fulda M, Shockey J, Werber M, Wolter FP, Heinz E (2002). Two long-chain acyl-CoA synthetases from Arabidopsis thaliana involved in peroxisomal fatty acid beta-oxidation. Plant J.

[35] Dormann P, Balbo I, Benning C (1999). Arabidopsis galactolipid biosynthesis and lipid trafficking mediated by DGD1. Science.

[36] Kelly AA, Froehlich JE, Dormann P (2003). Disruption of the two digalactosyldiacylglycerol synthase genes DGD1 and DGD2 in Arabidopsis reveals the existence of an additional enzyme of galactolipid synthesis. Plant Cell.

[37] Carrie C, Murcha MW, Millar AH, Smith SM, Whelan J (2007). Nine 3-ketoacyl-CoA thiolases (KATs) and acetoacetyl-CoA thiolases (ACATs) encoded by five genes in Arabidopsis thaliana are targeted either to peroxisomes or cytosol but not to mitochondria. Plant Mol Biol.

[38] Yu B, Wakao S, Fan J, Benning C (2004). Loss of plastidic lysophosphatidic acid acyltransferase causes embryo-lethality in Arabidopsis. Plant Cell Physiol.

[39] Li Z, Huang T, Tang M, Cheng B, Peng Y, Zhang X (2019). iTRAQ-based proteomics reveals key role of gamma-aminobutyric acid (GABA) in regulating drought tolerance in perennial creeping bentgrass (Agrostis stolonifera). Plant Physiol Biochem.

[40] Li F, Wu X, Lam P, Bird D, Zheng H, Samuels L, Jetter R, Kunst L (2008). Identification of the wax ester synthase/acyl-coenzyme a: diacylglycerol acyltransferase WSD1 required for stem wax ester biosynthesis in Arabidopsis. Plant Physiol.

[41] Jolivet P, Roux E, D'Andrea S, Davanture M, Negroni L, Zivy M, Chardot T (2004). Protein composition of oil bodies in Arabidopsis thaliana ecotype WS. Plant Physiol Biochem.

[42] Chen M, Xu R, Ji H, Greening DW, Rai A, Izumikawa K, Ishikawa H, Takahashi N, Simpson RJ (2016). Transcriptome and long noncoding RNA sequencing of three extracellular vesicle subtypes released from the human colon cancer LIM1863 cell line. Sci Rep.

[43] Wei S, Ma X, Pan L, Miao J, Fu J, Bai L, Zhang Z, Guan Y, Mo C, Huang H (2017). Transcriptome Analysis of Taxillusi chinensis (DC.) Danser Seeds in Response to Water Loss. PLoS One.

[44] Bryant DM, Johnson K, DiTommaso T, Tickle T, Couger MB, Payzin-Dogru D, Lee TJ, Leigh ND, Kuo TH, Davis FG, Bateman J, Bryant S, Guzikowski AR, Tsai SL, Coyne S, Ye WW, Freeman RM, Peshkin L, Tabin CJ, Regev A, Haas BJ, Whited JL (2017). A tissue-mapped axolotl De novo Transcriptome enables identification of limb regeneration factors. Cell Rep.

[45] Lagesen K, Hallin P, Rodland EA, Staerfeldt HH, Rognes T, Ussery DW (2007). RNAmmer: consistent and rapid annotation of ribosomal RNA genes. Nucleic Acids Res.

[46] Finn RD, Clements J, Eddy SR (2011). HMMER web server: interactive sequence similarity searching. Nucleic Acids Res.

[47] Petersen TN, Brunak S, von Heijne G, Nielsen H (2011). SignalP 4.0: discriminating signal peptides from transmembrane regions. Nat Methods.

[48] Krogh A, Larsson B, von Heijne G, Sonnhammer EL (2001). Predicting transmembrane protein topology with a hidden Markov model: application to complete genomes. J Mol Biol.

[49] Kanehisa M (2019). Toward understanding the origin and evolution of cellular organisms. Protein Sci.

[50] Chen M, Mithraprabhu S, Ramachandran M, Choi K, Khong T, Spencer A (2019). Utility of circulating cell-free RNA analysis for the characterization of global Transcriptome profiles of multiple myeloma patients. Cancers.

