# Supplementary material for: Dynamic transcriptome analysis identifies genes related to fatty acid biosynthesis in the seeds of Prunus pedunculata Pall
Source: BMC Plant Biol. 2021 Mar 24;21:152. doi: 10.1186/s12870-021-02921-x (PMC7992973; doi:10.1186/s12870-021-02921-x)
Supplement: Supplementary file 3 — Additional file 3. Numbers of differentially expressed genes of fatty acid related pathway/biological process. [file 12870_2021_2921_MOESM3_ESM.pdf]

Dynamic transcriptome analysis identifies genes related to fatty acid biosynthesis in the seeds of *Prunus pedunculata* Pall

Wenquan Bao[1], Dun Ao[1], Lin Wang[2*], Zhihao Ling[3], Maoshan Chen[4], Yue Bai[1], Ta-Na Wuyun[2], Junxing Chen[1], Shuning Zhang[1], Fengming Li[1]

[1]Inner Mongolia Agricultural University, Hohhot, China 010018

[2]State Key Laboratory of Tree Genetics and Breeding, Non-timber Forest Research and Development Center, Chinese Academy of Forestry, Zhengzhou, China 450003

[3]Chengdu JiYu Technology, Chengdu, Sichuan, China 610213

[4]Australian Center for Blood Diseases, Central Clinical School, Monash University, Melbourne, Victoria, Australia 3004

Corresponding author: Dr. Lin Wang (wanglin1815@163.com)

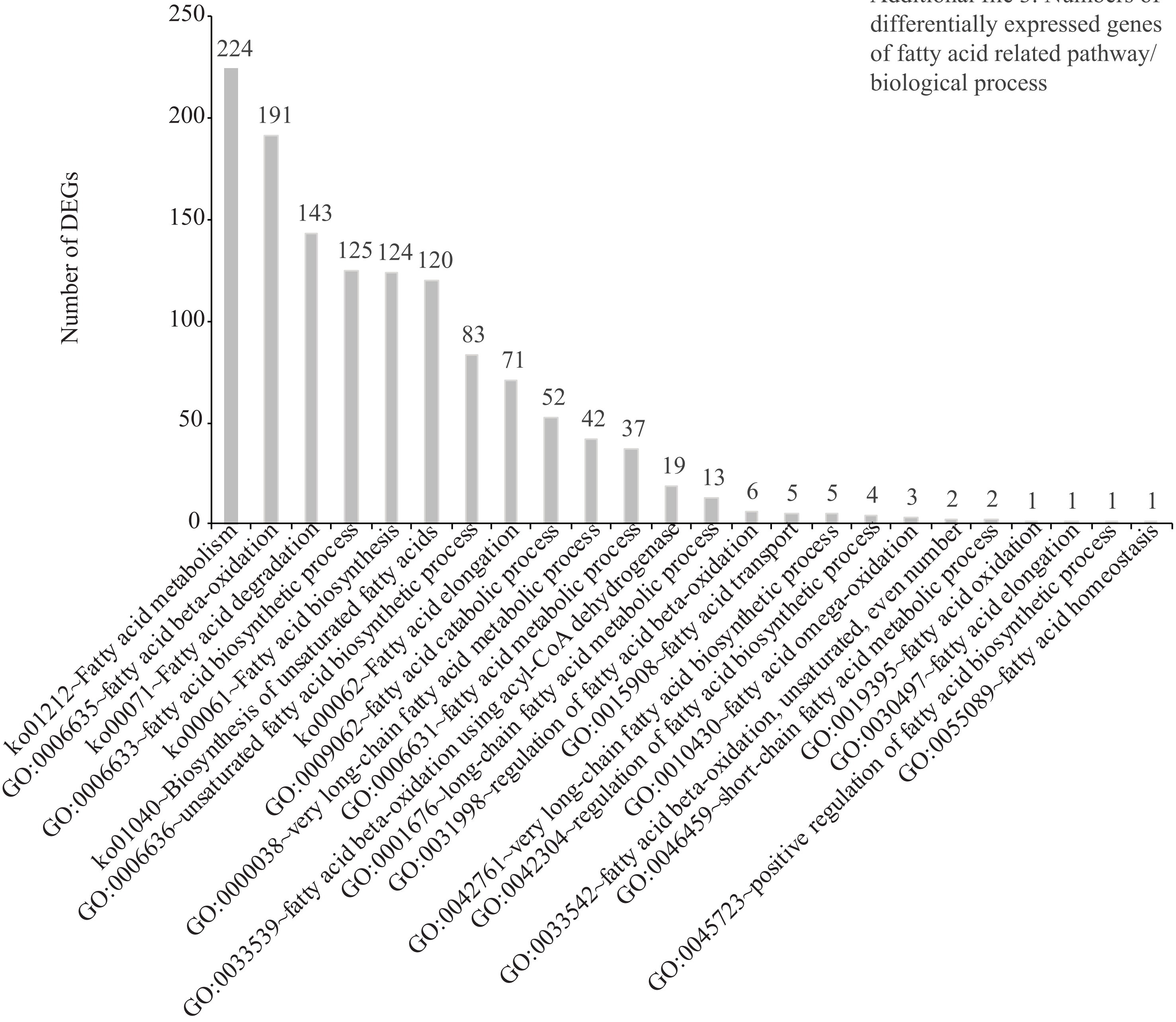
Additional file 3. Numbers of differentially expressed genes of fatty acid related pathway/ biological process
Number of DEGs
250
200
150
100
50
0
224
191
143
125
124
120
83
71
52
42
37
19
13
6
5
5
4
3
2
2
1
1
1
1
ko01212~Fatty acid metabolism
GO:0006635~fatty acid beta-oxidation
ko00071~Fatty acid degradation
GO:0006633~fatty acid biosynthetic process
ko00061~Fatty acid biosynthesis
ko01040~Biosynthesis of unsaturated fatty acids
GO:0006636~unsaturated fatty acid biosynthetic process
ko00062~Fatty acid elongation
GO:0009062~fatty acid catabolic process
GO:0000038~very long-chain fatty acid metabolic process
GO:0006631~fatty acid metabolic process
GO:0033539~fatty acid beta-oxidation using acyl-CoA dehydrogenase
GO:0001676~long-chain fatty acid metabolic process
GO:0031998~regulation of fatty acid beta-oxidation
GO:0015908~fatty acid transport
GO:0042761~very long-chain fatty acid biosynthetic process
GO:0042304~regulation of fatty acid biosynthetic process
GO:0010430~fatty acid omega-oxidation
GO:0033542~fatty acid beta-oxidation, unsaturated, even number
GO:0046459~short-chain fatty acid metabolic process
GO:0019395~fatty acid oxidation
GO:0030497~fatty acid elongation
GO:0045723~positive regulation of fatty acid biosynthetic process
GO:0055089~fatty acid homeostasis
